# Experimental Study on Impact-Induced Reaction Characteristics of PTFE/Ti Composites Enhanced by W Particles

**DOI:** 10.3390/ma10020175

**Published:** 2017-02-13

**Authors:** Yan Li, Zaicheng Wang, Chunlan Jiang, Haohao Niu

**Affiliations:** State Key Laboratory of Explosion Science and Technology, Beijing Institute of Technology, Beijing 100081, China; 3120130104@bit.edu.cn (Y.L.); wangskyshark@bit.edu.cn (Z.W.); 2120140238@bit.edu.cn (H.N.)

**Keywords:** energetic structural materials, PTFE/Ti/W composites, impact ignition, reaction threshold

## Abstract

Metal/fluoropolymer composites are a category of energetic structural materials that release energy through exothermic chemical reactions initiated under highly dynamic loadings. In this paper, the chemical reaction mechanism of PTFE (polytetrafluoroethylene)/Ti/W composites is investigated through thermal analysis and composition analysis. These composites undergo exothermic reactions at 510 °C to 600 °C, mainly producing TiF_x_. The tungsten significantly reduces the reaction heat due to its inertness. In addition, the dynamic compression properties and impact-induced reaction behaviors of PTFE/Ti/W composites with different W content prepared by pressing and sintering are studied using Split Hopkinson Pressure Bar and high speed photography. The results show that both the mechanical strength and the reaction degree are significantly improved with the increasing strain rate. Moreover, as W content increases, the mechanical strength is enhanced, but the elasticity/plasticity is decreased. The PTFE/Ti/W composites tend to become more inert with the increasing W content, which is reflected by the reduced reaction degree and the increased reaction threshold for the impact ignition.

## 1. Introduction

Metal/fluoropolymer composites have high energy density and rapidly release energy upon impact, which has allured researchers into intensely studying them in recent years [1,2]. Generally, metal/fluoropolymer composites are formed when active metal powders are uniformly mixed into a fluoropolymer matrix, cold pressed, and undergo a sintering hardening process to enhance their mechanical strength. These composites are sufficiently inert and insensitive to friction, flame, and detonation under normal conditions, which is markedly different from traditional energetic materials, such as explosives and propellants. Under intense impact, however, shock energy drives the active metal and the fluoropolymer to react violently, causing a detonation-like phenomenon to occur that releases a large amount of thermal energy and gaseous products. Due to their mechanical and energetic properties, metal/fluoropolymer composites are widely used in both military and civilian applications as replacements for components traditionally made of inert materials, such as fragmentation warheads, shaped-charge warheads, penetrating warheads, and oil-well perforations [3]. Unlike the mechanical perforation and kinetic energy damage caused by traditional inert metal projectiles, these energetic composites are initiated to deflagrate during penetration with enough velocity to result in significant structural damage to the targets.

Progress on active metal/fluoropolymer composites has advanced greatly in recent years, especially in shock-induced chemical reactions. The experimental approaches used are commonly based on impact loading, which can be classified as direct impact, indirect impact, or two-step impact [4]. McGregor [5] performed plate impact tests to determine the reaction onset time of highly porous PTFE (polytetrafluoroethylene)/Al mixtures. Ames [6], Mock [7,8], and Shen [9] used Taylor impact tests to study the impact initiation of PTFE/Al and PTFE/Ti rods, establishing the relationship between ignition delay time and impact energy. Moreover, a vented test chamber was designed to measure the energy release of reactive fragments [10,11,12,13], with results showing impact velocity, strength properties, and reactive material formulation each have significant influence on energy release behavior. Dolgoborodov [14] researched the combustion propagation in highly porous PTFE/Al mixtures initiated by low detonation velocity explosives, and proved that it is possible to achieve steady detonation.

However, compared to conventional metals and alloys, active metal/fluoropolymer composites, such as PTFE/Al and PTFE/Ti, have relatively low densities and material strength properties. Therefore, tungsten (W) particles are typically added to the composites to increase density and structural strength. Recently, researchers have carried out studies on the reaction characteristics of active metal/fluoropolymer composites enhanced by W particles. Herbold [15] researched the shock behavior of PTFE/Al/W granular composites through numerical analysis and demonstrated that a higher thermal energy of the PTFE and Al can be reached with the increase of W particles, which may be important for igniting the reaction. Xu [16] performed a pendulum impact test to investigate the reaction behaviors of PTFE/Al/W composites and revealed that the addition of W particles decreases the reaction activity and completeness. Wang [17] applied Hopkinson bar techniques to investigate the impact insensitivity of PTFE/Al/W composites with different W percentages and determined that the ignition delay time, energy absorbed before the reaction, and incompleteness all exhibit clear increases with increasing W content. However, the aforementioned research paid little attention to the strain-rate and energy threshold of the impact-induced reaction or to the influence of W content on the reaction threshold, all of which are extremely important for the applications of energetic structural materials.

This paper presents research on the impact-induced reaction characteristics of PTFE/Ti composites enhanced by W particles. The Split-Hopkinson Pressure Bar (SHPB) technique was used to study PTFE/Ti/W composites with different W contents. The strain-rate threshold, unit energy threshold, ignition delay time, and reaction duration were investigated and the influence of W content on these reaction characteristics was elucidated. Further, the chemical reaction mechanism and dynamic compression properties of PTFE/Ti/W composites were established.

## 2. Experimental Materials

### 2.1. Preparation of Experimental Samples

In this study, four PTFE/Ti/W composites with mass ratios of 68/32/0, 47/23/30, 34/16/50, and 20/10/70 were used. The relative mass ratio of PTFE to Ti was determined according to stoichiometry. W particles were added in varying amounts, making the composites reach varying densities. Initially, the powders had the following average particle sizes: PTFE 34 μm, Ti 30 μm, and W 40 μm.

**Figure 1:**
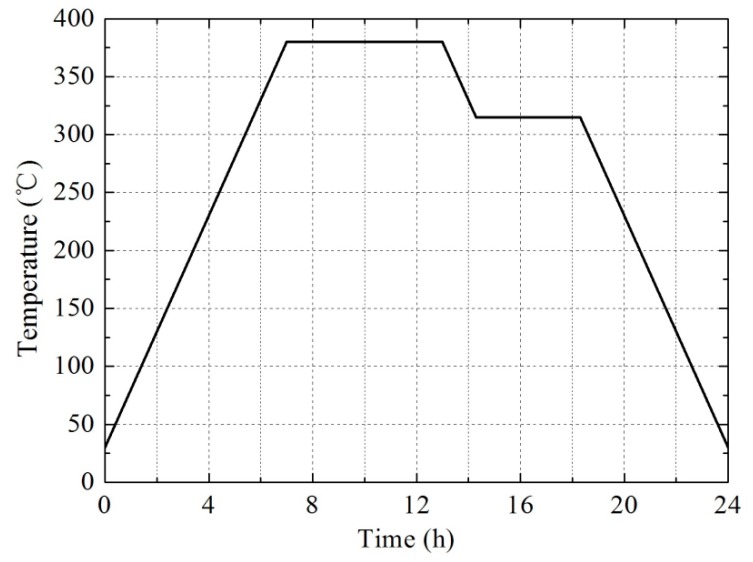
The temperature steps of a sintering cycle.

The preparation process for PTFE/Ti/W composites is based on Nielson, et al.’s patent [18], which can be described as follows:

(1) First, the PTFE, Ti, and W powders were mixed with a small amount of absolute alcohol as a medium for 10 h by a planetary mill machine. Then the powders were dried at 57.2 °C in a vacuum drying oven for approximately 24 h.
(2) The dried powder mixtures were pressed at 200 MPa for about 3 min through cold uniaxial pressing, and the cylindrical billets were prepared.
(3) The pressed billets were relaxed at ambient pressure and temperature for 24 h to remove any trapped air or residual stress, and then they were sintered in an argon atmosphere at 380 °C. Figure 1 shows the temperature steps of the sintering cycle, which can be described as follows: The oven temperature was raised up to 380 °C at a rate of about 50 °C/h. The billets were held at 380 °C for 6 h, then the temperature was reduced at a rate of about 50 °C/h to 315 °C and maintained for 4 h. The billets were then cooled to ambient temperature at an average cooling rate of 50 °C/h.
(4) Finally, the sintered billets were processed into cylindrical experimental samples by a milling machine with a final size of φ 8 × 5 mm^2^.

**Figure 2:**
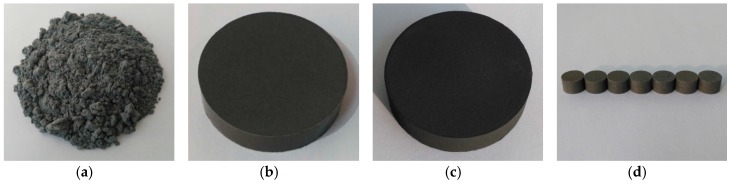
Photographs of PTFE/Ti/W composites at various preparation stages: (**a**) powder mixture; (**b**) pressed billet; (**c**) sintered billet; (**d**) experimental samples.

Photographs of typical powder mixture, pressed billet, sintered billet, and experimental samples are shown in Figure 2. Table 1 presents the theoretical maximum density (TMD) of the four PTFE/Ti/W composites, along with the corresponding actual and relative density after preparation.

### 2.2. Microstructure of Composites

**Figure 3:**
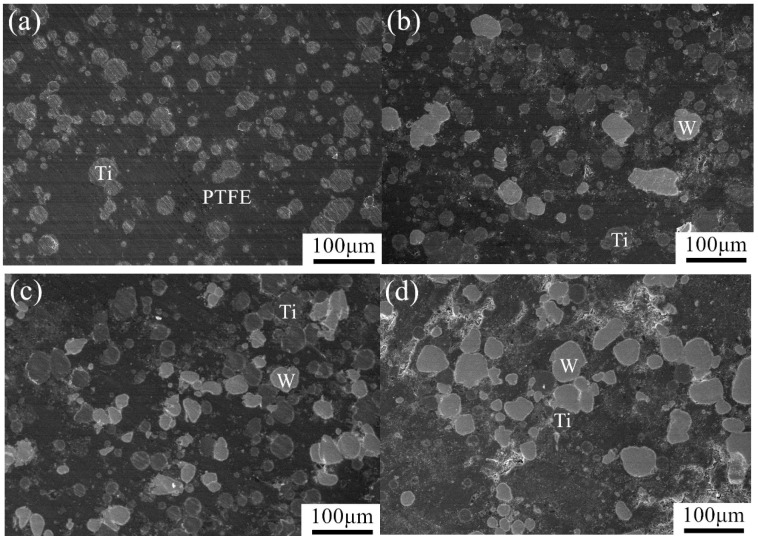
Backscattered scanning electron microscope (SEM) images of PTFE/Ti/W composites: (**a**) 68PTFE/32Ti; (**b**) 47PTFE/23Ti/30W; (**c**) 34PTFE/16Ti/50W; (**d**) 20PTFE/10Ti/70W.

The microstructures of the PTFE/Ti/W composites were determined using backscattered scanning electron microscopy under 800 times magnification, as shown in Figure 3. These images demonstrate that the PTFE forms a continuous matrix in which W and Ti particles are discretely distributed. The spherical W particles appear as relatively bright features in the backscattered images because W has a high atomic number. The Ti agglomerates are approximately spherical and have a grey level between that of PTFE and W.

### 2.3. Reaction Mechanism Analysis

**Figure 4:**
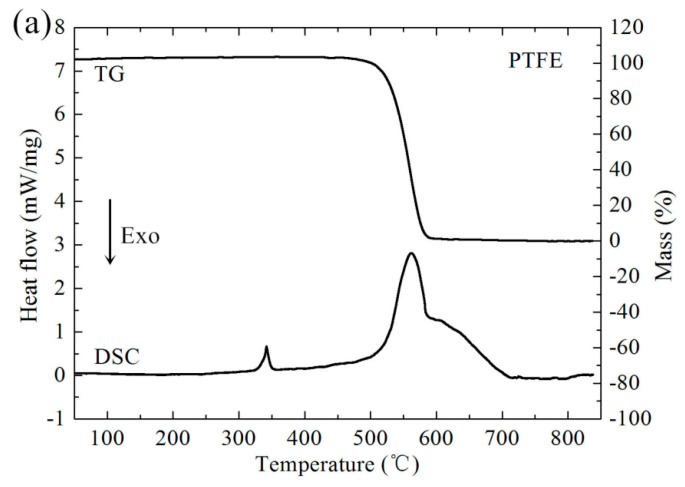

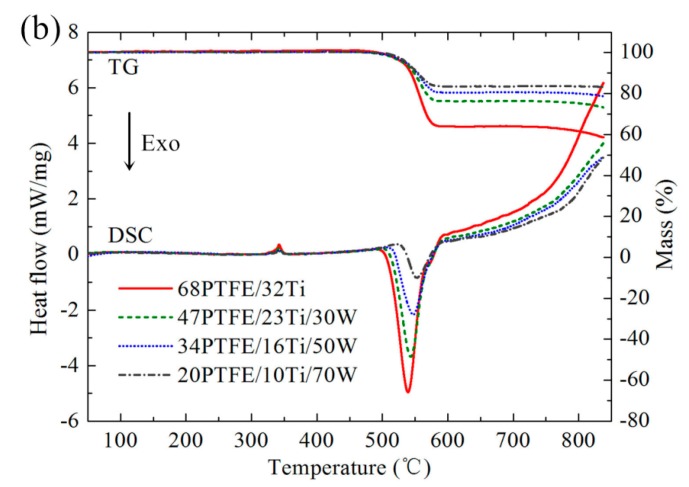
DSC/TG curves of (**a**) Pure PTFE and (**b**) PTFE/Ti/W composites at 5 °C/min heating rate.

**Figure 5:**
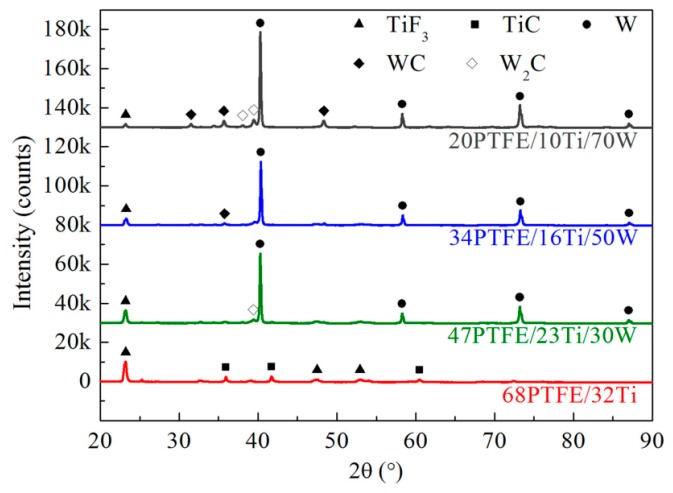
XRD results of solid reaction products of PTFE/Ti/W composites after DSC tests.

Before conducting Split Hopkinson Pressure Bar (SHPB) experiments, the chemical reaction mechanism of PTFE/Ti/W composites was investigated. Pure PTFE, 68PTFE/32Ti, 47PTFE/23Ti/30W, 34PTFE/16Ti/50W, and 20PTFE/10Ti/70W were each analyzed by Differential Scanning Calorimetry (DSC) and Thermogravimetry (TG), which were performed at the same time on the same sample from room temperature to 850 °C in argon atmosphere. The heating rate was 5 °C/min. The post-DSC/TG solid reaction products of all the PTFE/Ti/W composites were analyzed by X-ray Diffraction (XRD). Figure 4 shows the DSC and TG curves. Figure 5 shows the XRD results.

Pure PTFE melts at around 342 °C with a small endothermic peak, which is followed by a strongly endothermic decomposition process starting at 400 °C with the peak at 562 °C, as shown in Figure 4. The corresponding mass drops to zero due to the phase transition of condensed PTFE to gaseous C_2_F_4_. PTFE/Ti undergoes an exothermic reaction from 510 to 600 °C with the peak at 539 °C, which overlaps the temperature range of PTFE decomposition. At this stage, Ti is oxidized by C_2_F_4_, producing TiF_3_ and a small amount of TiC, as shown in the XRD patterns. Moreover, TiF_4_ is also likely to be generated because it is more chemically stable than TiF_3_. However, TiF_4_ is not detected in the XRD patterns due to its volatilization. When the temperature rises to 740 °C, the mass is further reduced, which may be due to the disproportion reaction of TiF_3_, generating solid Ti and gaseous TiF_4_. The 47PTFE/23Ti/30W, 34PTFE/16Ti/50W, and 20PTFE/10Ti/70W composites have DSC/TG curves with features similar to that of PTFE/Ti, but the exothermic peak, peak area, and mass loss extent are significantly reduced with increasing W contents. Furthermore, W produces an intense diffraction peak in the XRD patterns, and only a small amount of W is converted to WC or W_2_C, proving W is insensitive to the temperature test range. In summary, the possible chemical reactions within PTFE/Ti/W composites in argon atmosphere can be concluded as follows:

(-C_2_F_4_-) → C_2_F_4_ (g)

4Ti + 3C_2_F_4_ → 4TiF_3_ (s) + 6C

Ti + C_2_F_4_ → TiF_4_ (g) + 2C

Ti + C → TiC

4TiF_3_ → 3TiF_4_ (g) + Ti

W + C → WC

2W + C → W_2_C

The reactions between Ti and C_2_F_4_ release a large amount of energy, so they are considered the main chemical reactions. The addition of inert W powder reduces the amount of PTFE/Ti reactant present in the composites, resulting in decreased reaction heat per unit of mass, which can be visualized by the reduction of the exothermic peak area.

## 3. SHPB Experiments

**Figure 6:**
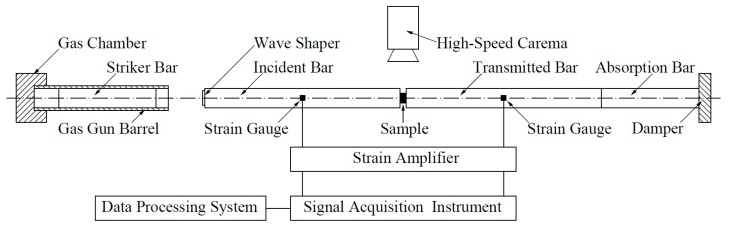
Schematic diagram of the SHPB test system.

Dynamic impact testing was performed using the Split Hopkinson Pressure Bar technique, which comprises of four 16 mm-diameter steel bars: a striker bar, an incident bar, a transmitted bar, and an absorption bar. Figure 6 shows the SHPB test system. A φ 8 × 5 mm^2^ sample is sandwiched between the incident and transmitted bars. When the striker bar hits the incident bar, an elastic pulse wave is generated and propagates in the bars, causing the sample to be compressed. Strain gauges are attached to the bars to measure the strain pulse, from which the dynamic compression response of the sample is obtained. A high-speed camera is also used to observe the impact-induced reaction phenomenon of PTFE/Ti/W composites.

The SHPB test is based on the assumption that the stress and deformation throughout the sample is homogenous and uniform. PTFE/Ti/W composites have relatively low strength and mechanical impedance compared to conventional metals and alloys, which may result in a non-equilibrium state during compression. To circumvent this problem, a pulse shaper is attached to the incident bar to reduce the initial slope of the incident pulse as well as decrease the dispersion effects of the signals, and bring the sample near a state of dynamic stress equilibrium.

## 4. Results and Discussion

### 4.1. Dynamic Compression Properties

**Figure 7:**
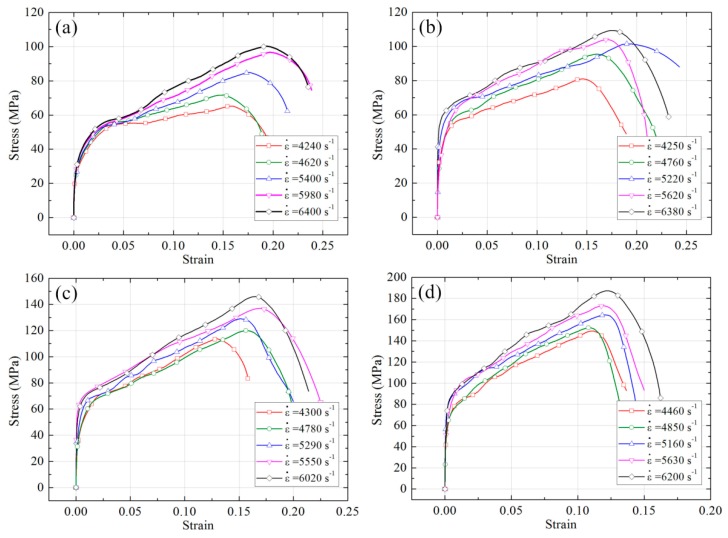
True stress-strain curves of PTFE/Ti/W composites under dynamic compression: (**a**) 68PTFE/32Ti; (**b**) 47PTFE/23Ti/30W; (**c**) 34PTFE/16Ti/50W; (**d**) 20PTFE/10Ti/70W.

The true stress-strain curves of four PTFE/Ti/W composites under dynamic compression are shown in Figure 7, and the corresponding yield strength, compressive strength, and critical failure strain at different strain rates are listed in Table 2.

The dynamic compression response of the PTFE/Ti/W composites can be divided into three stages: elastic stage, plastic stage, and damage stage. A significant strain hardening effect can be observed for all the composites. The yield strength, compressive strength, and critical failure strain all improve with the increasing strain rate, showing an obvious strain rate effect. Moreover, as W content increases, both the yield strength and compressive strength are improved, meaning W particles impart a significant mechanical enhancement effect. However, the critical failure strain as well as the nonlinear degree of elastic deformation is reduced, indicating the PTFE/Ti/W composites tend to become more brittle with increased W content.

**Figure 8:**
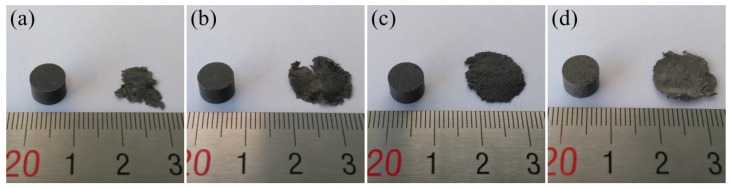
Samples before and after SHPB tests of PTFE/Ti/W composites: (**a**) 68PTFE/32Ti; (**b**) 47PTFE/23Ti/30W; (**c**) 34PTFE/16Ti/50W; (**d**) 20PTFE/10Ti/70W.

**Figure 9:**
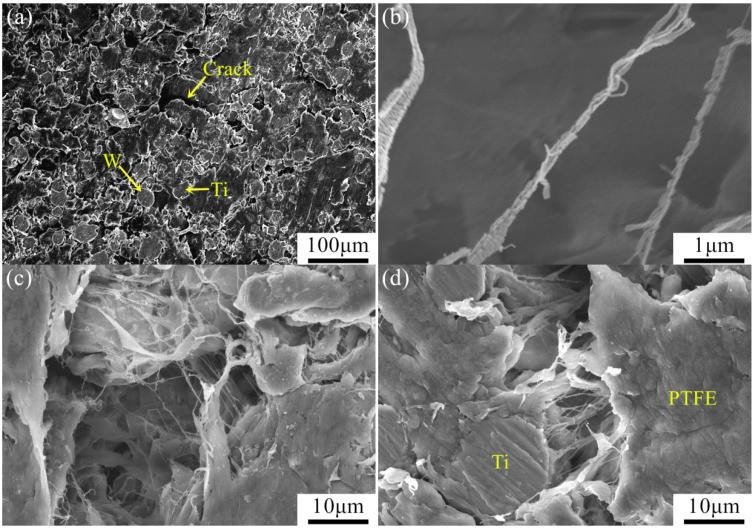
SEM micrographs of the sample residues: (**a**) overall view of sample surface; (**b**) enlarged view of PTFE nano-fibers; (**c**) network of PTFE nano-fibers; (**d**) crack in the metal/matrix interface and PTFE fibers connecting to metal particle.

PTFE/Ti/W samples before and after SHPB tests are shown in Figure 8. Sheet features are present in all of the samples after impact tests. The mass of the samples is obviously decreased due to fragmentation and chemical reactions initiated by impact. SEM micrographs of the unreacted sample residues are shown in Figure 9. Both the Ti and W particles lack any visible deformation, while the PTFE matrix is stretched, forming cracks and fibers along the stretching direction. An enlarged view shows that the fibers are as narrow as 50–200 nm in diameter. The nano-fibers interweave with each other, generating a dense network and providing resistance for the propagating crack. Cracks are also formed in the metal/matrix interfaces, where the PTFE fibers are connected to the metal particles. Fracture of the PTFE matrix and the separation of metal particles from the matrix are therefore speculated to be the major mechanisms leading to failure of the samples [19,20].

### 4.2. Impact-Induced Reaction Characteristics

**Figure 10:**
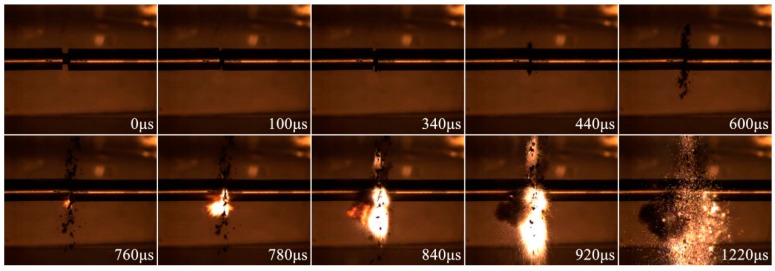
Video frames of 68PTFE/32Ti composites under 5980 s^−1^ strain rate captured from various time points.

The deformation, failure and reaction of PTFE/Ti/W composites under dynamic compression were recorded by the high-speed camera with a frame rate of 50,000 fps. Frames of 68PTFE/32Ti composites under 5980 s^−1^ strain rate from various time points are shown in Figure 10. The time of impact is set as 0 μs. The sample is intensely compressed in the initial 100 μs with a continuous deformation. A small quantity of debris is jettisoned at about 100 μs, when the maximum stress in the sample is reached and the failure of the sample occurs. During the following 240 μs, little deformation of the sample is observed. At this stage, the cracks are generated and quickly propagated, followed by a sudden fracture, producing a large amount of debris due to the widely distributed cracks in the sample. At 760 μs, the reaction is initiated. Firelight is produced, increases, and eventually disappears after about 1220 μs. Thus, the impact-induced chemical reaction happens at some time after the failure of the sample. The reaction has an ignition delay time.

**Figure 11:**
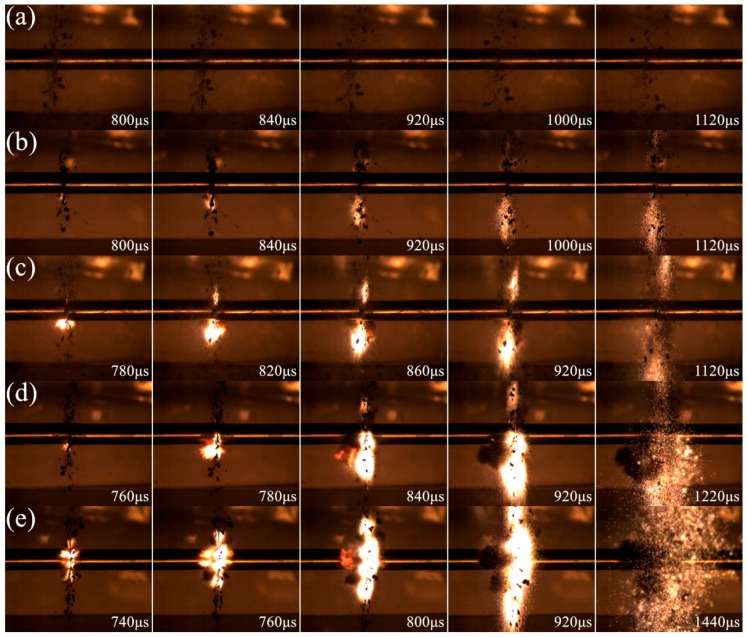
Video frames of 68PTFE/32Ti composites captured under different strain rates: (**a**) 4240 s^−1^; (**b**) 4620 s^−1^; (**c**) 5400 s^−1^; (**d**) 5980 s^−1^; (**e**) 6400 s^−1^.

The impact-induced reaction process of 68PTFE/32Ti composites at five different strain rates is presented in Figure 11. Little firelight is produced at the 4240 s^−1^ strain rate. However, at the strain rates from 4620 to 6400 s^−1^, the reaction is initiated with varying degrees of firelights, and the reaction is significantly enhanced with the increasing strain rate, as shown in Figure 11b–e. The initial time of the reaction delays from 800 to 740 μs, and the reaction duration varies from roughly 320 to 700 μs, proving that strain rate plays a significant role in the impact-induced chemical reaction, and there should be a critical strain rate below which no reaction will occur.

**Figure 12:**
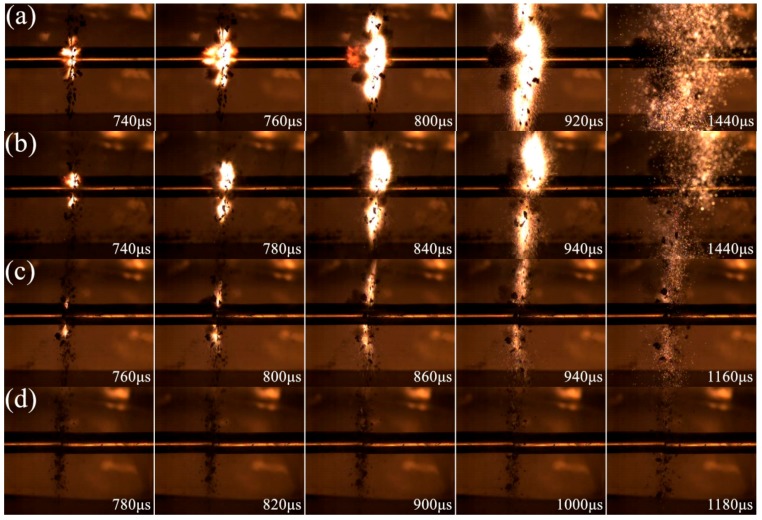
Video frames of PTFE/Ti/W composites with different W content under the similar strain rates: (**a**) 68PTFE/32Ti at 6400 s^−1^; (**b**) 47PTFE/23Ti/30W at 6380 s^−1^; (**c**) 34PTFE/16Ti/50W at 6020 s^−1^; (**d**) 20PTFE/10Ti/70W at 6200 s^−1^.

The impact-induced reaction process of PTFE/Ti/W composites with varying W content under the similar strain rate is depicted in Figure 12. The W particles, which act as a mechanical enhancement factor, do not participate in the reaction due to their high chemical stability. Though a higher thermal energy of PTFE and Ti may be reached with the increasing W content due to the extrusion and friction caused by rigid W particles according to Herbold’s simulation results [15], the quantity of reactant is decreased when inert W particles are added. Moreover, the contact area of active Ti and PTFE may be reduced due to the separation caused by W particles, and some of the impact energy is absorbed by the W particles, both of which are disadvantageous for the reaction. Therefore, as W content increases, the reaction degree is significantly reduced, ignition delay time presents an increasing trend, and reaction duration gradually decreases. Little firelight is displayed for 20PTFE/10Ti/70W composites because of the highest W content. A higher strain rate would be necessary if the reaction could still be initiated. The reaction characteristics of PTFE/Ti/W composites with varying strain rate and W content are listed in Table 3.

During dynamic impact, the sample absorbs the mechanical work of the bars, and converts this work into the sample’s internal energy. When the local temperature rises to the ignition temperature, the reaction is initiated and spreads through the sample. The thermodynamic process can be considered adiabatic due to the extremely short duration. The absorbed energy per unit volume of the sample can be expressed by the sample’s strain energy, which is calculated as follows:

$$E=\int_{0}^{\varepsilon_{m}}\sigma (\varepsilon )d\varepsilon$$

where *E* is the specific energy, $\varepsilon_{m}$ is the failure strain, σ is the stress, and ε is the strain. From this equation, the specific energy can be interpreted as the area of the stress-strain curve and the abscissa. When the strain value is greater than the failure strain, fracture of the sample occurs, and the stress drops sharply. In this stage, the strain energy can be ignored.

In this experiment, the strain rate threshold is considered the strain rate below which the reaction will not occur. The specific energy threshold is defined as the specific energy absorbed by the sample at the critical strain rate. The reaction thresholds for impact ignition of PTFE/Ti/W composites are listed in Table 4.

As can be seen from Table 4, both the strain rate and the specific energy thresholds are improved with increasing W content: the strain rate threshold increases from 4620 to over 6200 s^−1^, and the specific energy threshold increases from 9.5 to over 19.7 J/cm^3^. The addition of inert W particles improves the density and the mechanical strength of PTFE/Ti/W composites, but also reduces the reactive activity. As the W content increases, PTFE/Ti/W composites require increased strain rate and impact energy to initiate the reaction.

## 5. Conclusions

In this study, four PTFE/Ti/W composites were prepared. The reaction mechanisms of these composites was analyzed. Moreover, the effects of strain rate and W content on mechanical strength and reaction characteristics were elucidated through the SHPB technique. The following specific conclusions can be drawn from the results:

(1) PTFE/Ti/W composites undergo exothermic reactions at 510 °C to 600 °C, which overlaps the temperature range of PTFE decomposition. The major reaction product is TiF_x_, and little W undergoes any chemical change. The reaction heat is significantly reduced with increasing W content.
(2) PTFE/Ti/W composites exhibit obvious strain hardening effect and strain rate effect under dynamic compression. Mechanical strength is improved as W content increases, whereas the failure strain is reduced. The PTFE/Ti/W composites tend to become more brittle with the increasing W content. The major failure mechanisms of the composites are the fracture of the PTFE matrix and the separation of metal particles from the matrix.
(3) The impact-induced reaction of PTFE/Ti/W composites occurs at some time after the material failure. Increasing the strain rate significantly improves the reaction degree. As W content increases, the reaction degree is greatly reduced. The ignition delay time becomes longer, while the reaction duration becomes shorter. Both the strain rate and the specific energy thresholds for impact ignition are improved. PTFE/Ti/W composites tend to become more inert with increasing W content.

## Figures and Tables

**Table 1:** Densities of the PTFE/Ti/W composites.

Composites	TMD (g/cm^3^)	Density (g/cm^3^)	Relative Density
PTFE/Ti/W (68/32/0)	2.64	2.60	98.5%
PTFE/Ti/W (47/23/30)	3.56	3.48	97.8%
PTFE/Ti/W (34/16/50)	4.65	4.53	97.4%
PTFE/Ti/W (20/10/70)	6.67	6.44	96.6%

**Table 2:** Mechanical parameters of PTFE/Ti/W composites at different strain rates

Composites	Strain Rate (s^−1^)	Yield Strength (MPa)	Compressive Strength (MPa)	Failure Strain
PTFE/Ti/W (68/32/0)	4240	45.1	65.2	0.15
	4620	46.2	71.7	0.15
	5400	47.6	84.8	0.18
	5980	49.8	96.7	0.20
	6400	50.8	100.3	0.19
PTFE/Ti/W (47/23/30)	4250	55.9	80.9	0.14
	4760	50.6	95.5	0.16
	5220	66.0	101.4	0.19
	5620	64.4	104.0	0.17
	6380	68.2	109.3	0.18
PTFE/Ti/W (34/16/50)	4300	70.0	113.7	0.13
	4780	65.0	120.1	0.18
	5290	70.6	129.0	0.15
	5550	77.2	136.9	0.17
	6020	76.0	145.9	0.17
PTFE/Ti/W (20/10/70)	4460	88.8	149.2	0.11
	4850	93.6	152.3	0.11
	5160	103.5	164.5	0.12
	5630	106.2	173.0	0.12
	6200	105.8	187.1	0.12

**Table 3:** Ignition delay time and reaction duration of PTFE/Ti/W composites.

Composites	Strain Rate (s^−1^)	Ignition Delay Time (μs)	Reaction Duration (μs)
PTFE/Ti/W (68/32/0)	4240	No reaction	-
	4620	800	320
	5400	780	340
	5980	760	460
	6400	740	700
PTFE/Ti/W (47/23/30)	4250	No reaction	-
	4760	No reaction	-
	5220	800	300
	5620	760	440
	6380	740	700
PTFE/Ti/W (34/16/50)	4300	No reaction	-
	4780	No reaction	-
	5290	No reaction	-
	5550	780	340
	6020	760	400
PTFE/Ti/W (20/10/70)	4460	No reaction	-
	4850	No reaction	-
	5160	No reaction	-
	5630	No reaction	-
	6200	No reaction	-

**Table 4:** Strain rate thresholds and specific energy thresholds of PTFE/Ti/W composites.

Composites	Strain Rate Threshold (s^−1^)	Specific Energy Threshold (J/cm^3^)
PTFE/Ti/W (68/32/0)	4620	9.5
PTFE/Ti/W (47/23/30)	5220	15.3
PTFE/Ti/W (34/16/50)	5550	17.8
PTFE/Ti/W (20/10/70)	>6200	>19.7
